# The Effects of Using the Sun Safe App on Sun Health Knowledge and Behaviors of Young Teenagers: Results of Pilot Intervention Studies

**DOI:** 10.2196/35137

**Published:** 2022-03-16

**Authors:** Isabelle M Clare, Nisali Gamage, Gail A Alvares, Lucinda J Black, Jacinta Francis, Mohinder Jaimangal, Robyn M Lucas, Mark Strickland, James White, Rebecca Nguyen, Shelley Gorman

**Affiliations:** 1 Telethon Kids Institute University of Western Australia Perth Australia; 2 Curtin School of Population Health Curtin University Perth Australia; 3 Curve Tomorrow Perth Australia; 4 National Centre for Epidemiology and Population Health Research School of Population Health Australian National University Canberra Australia; 5 Centre for Ophthalmology and Visual Science University of Western Australia Perth Australia; 6 Cancer Council Western Australia Perth Australia; 7 Reach Health Promotion Innovations Perth Australia

**Keywords:** app development, co-design, knowledge gain, sun exposure, sun protection, sun behaviors, teenagers, UV Index, vitamin D, young adolescents, mobile phone

## Abstract

**Background:**

A balanced approach toward sun exposure and protection is needed by young people. Excessive sun exposure increases their risk for skin cancers such as melanoma, whereas some exposure is necessary for vitamin D and healthy bones. We have developed a new iOS smartphone app—Sun Safe—through a co-design process, which aims to support healthy and balanced decision-making by young teenagers (aged 12-13 years).

**Objective:**

The aim of this study was to test the capacity of Sun Safe to improve sun health knowledge and behaviors of young teenagers in 3 pilot intervention studies completed in 2020.

**Methods:**

Young teenagers (aged 12-13 years; N=57) were recruited through the web or through a local school via an open-access website and given access to Sun Safe (29/57, 51%) or a placebo (SunDial) app (28/57, 49%). Participants completed sun health questionnaires and knowledge quizzes before and after the 6-week intervention (either on the web or in class) and rated the quality of the app they used via a survey.

**Results:**

Of the 57 participants, 51 (89%) participants (26, 51% for placebo arm and 25, 49% for the Sun Safe arm) completed these studies, with most (>50%) reporting that they used a smartphone to access their designated app either “once a fortnight” or “once/twice in total.” Improved sun health knowledge—particularly about the UV Index—was observed in participants who were given access to Sun Safe compared with those who used the placebo (−6.2 [percentage correct] difference in predicted means, 95% CI –12.4 to –0.03; *P*=.049; 2-way ANOVA). Unexpectedly, there were significantly more sunburn events in the Sun Safe group (relative risk 1.7, 95% CI 1.1-1.8; *P*=.02; Fisher exact test), although no differences in time spent outdoors or sun-protective behaviors were reported. COVID-19 pandemic–related community-wide shutdowns during April 2020 (when schools were closed) reduced the time spent outdoors by >100 minutes per day (–105 minutes per day difference in predicted means, 95% CI –150 to –59 minutes per day; *P*=.002; paired 2-tailed Student *t* test). Sun Safe was well-rated by participants, particularly for information (mean 4.2, SD 0.6 out of 5).

**Conclusions:**

Access to the Sun Safe app increased sun health knowledge among young teenagers in these pilot intervention studies. Further investigations with larger sample sizes are required to confirm these observations and further test the effects of Sun Safe on sun-protective behaviors.

## Introduction

### Sun Health Promotion and Behaviors: Australian Teenagers

A balanced approach toward sun protection and sun exposure is needed to promote the health and development of young people living in Australia. Sun-protective messaging aims to prevent sunburn and intermittent excessive sun exposure during childhood and adolescence as these events increase the risk for melanoma [1]. Conversely, some sun exposure is needed for vitamin D, healthy bone development, and other normal physiological and disease-preventing processes [2,3]. Although Australian teenagers have good knowledge about the importance of sun protection for preventing melanoma, they underestimate the risks associated with sunburn in childhood and adolescence [4]. Healthy sun behaviors are promoted in Australia through the entrenched *SunSmart* programs of the Cancer Council in primary (elementary) schools. However, these supportive programs are less well-established in secondary schools. This reduced support coincides with a time of life when *risky* behaviors emerge in young teenagers.

### Factors Affecting the Use of Sun Protection by Australian Teenagers

Other factors may also affect the use of sun protection by young people, including personal preference for tanned skin, peer influences, and resistance to adult advice [1,5,6]. Furthermore, communicating nuanced health messages about the fact that short regular exposures to sunlight are likely sufficient to maintain or raise circulating 25-hydroxyvitamin D levels (but insufficient to cause sunburn) [7] is challenging. Historical and existing health messaging in Australia has largely been via mass media (ie, news and television) campaigns of the Cancer Council. Novel approaches are emerging, such as the installation of highly visible UV meters in secondary schools [8]. Indeed, new public health strategies that target young adolescents are needed, which build on knowledge obtained from primary education and ongoing public health campaigns and provide more support to children as they transition into secondary schooling [9]. Currently, there is little specific mobile health support for the young adolescent age group, with more available for younger children (eg, *Cache-Cache Soliel* [10]), older teenagers (eg, *Sunface UV-selfie* [11]), and adults (eg, *SunSmart* [12]).

### The Sun Safe App is a Health Promotion e-Tool for Australian Teenagers

We recently co-developed an Apple iOS app—*Sun Safe*—with young teenagers (aged 12-13 years), Australian sun health promotion experts and researchers, and digital health developers [9]. The process underpinning the co-design of *Sun Safe* is reported in detail elsewhere [9]. This app aims to improve sun health knowledge and promote sun safe practices among young adolescents, including effective protection from sunburn and sufficient exposure for vitamin D. The health promotion message underlying *Sun Safe* is for users to *spend some time outdoors being active for vitamin D using sun protection as indicated by the UV Index*. The UV Index is a linear scale (1 to >11) of the intensity of solar UV radiation, categorized to describe the daily danger (from low to extreme) of sunburn. It is widely used by health promotion agencies around the world (including Cancer Councils Australia and the World Health Organization) to help people make decisions regarding sun protection.

### Study Objectives

Here, we report the findings of effectiveness pilot intervention studies that tested the capacity of *Sun Safe* to affect sun health knowledge and behaviors of young adolescents under *real-world* conditions. This research was conducted in 2020, with data collected across 3 pilot trials because of the impact of the COVID-19 pandemic (Multimedia Appendix 1) [13-32]. Our objectives are to obtain end user responses to *Sun Safe*, pilot-test its capacity to improve the sun health knowledge and behaviors of young adolescents (aged 12-13 years), estimate its likely acceptance and effectiveness, provide data to estimate sample sizes, and test recruitment strategies and methods for future definitive trials.

## Methods

Additional details on the methodology are provided in Multimedia Appendix 1.

### Ethical and Governance Approvals

Approval to conduct this study was obtained from the human research ethics committee of the University of Western Australia (WA; RA/4/20/4424). Project approval was received from the Department of Education of WA to allow researchers to recruit participants through a local Perth school [9]. Findings are reported according to CONSORT-EHEALTH (Consolidated Standards of Reporting Trials of Electronic and Mobile Health Applications and Online Telehealth) guidelines for pilot trials. This was a small pilot trial of a nonclinical intervention and not a randomized clinical trial.

### Timing of Pilot Intervention Studies

Parallel-designed, placebo-controlled pilot intervention studies were conducted across 2020, with participants recruited through community-based social media strategies or through a local high school (in class). Three pilot studies were conducted:

1. Community phase 1 pilot study (February 2020-May 2020)
2. School pilot study (February 2020-November 2020)
3. Community phase 2 pilot study (July 2020-November 2020)

### Recruitment of Participants

Recruitment was undertaken over two 5-week periods (February 2020 to March 2020 and July 2020 to August 2020). For community pilot studies, recruitment was conducted through notices placed on the Telethon Kids Facebook page (with >19,000 followers) and paid advertisements (total budget=Aus $400 [US $290]) specifically targeting parents living in WA aged ≥30 years. In the school pilot study, participants were recruited via in-class sessions with researchers speaking to 3 classes of students in years 7 and 8. Please see the *Methods* section of Multimedia Appendix 1 for COVID-19 pandemic impacts on recruitment and more details regarding timelines.

### Eligibility Criteria

**Figure 1:**
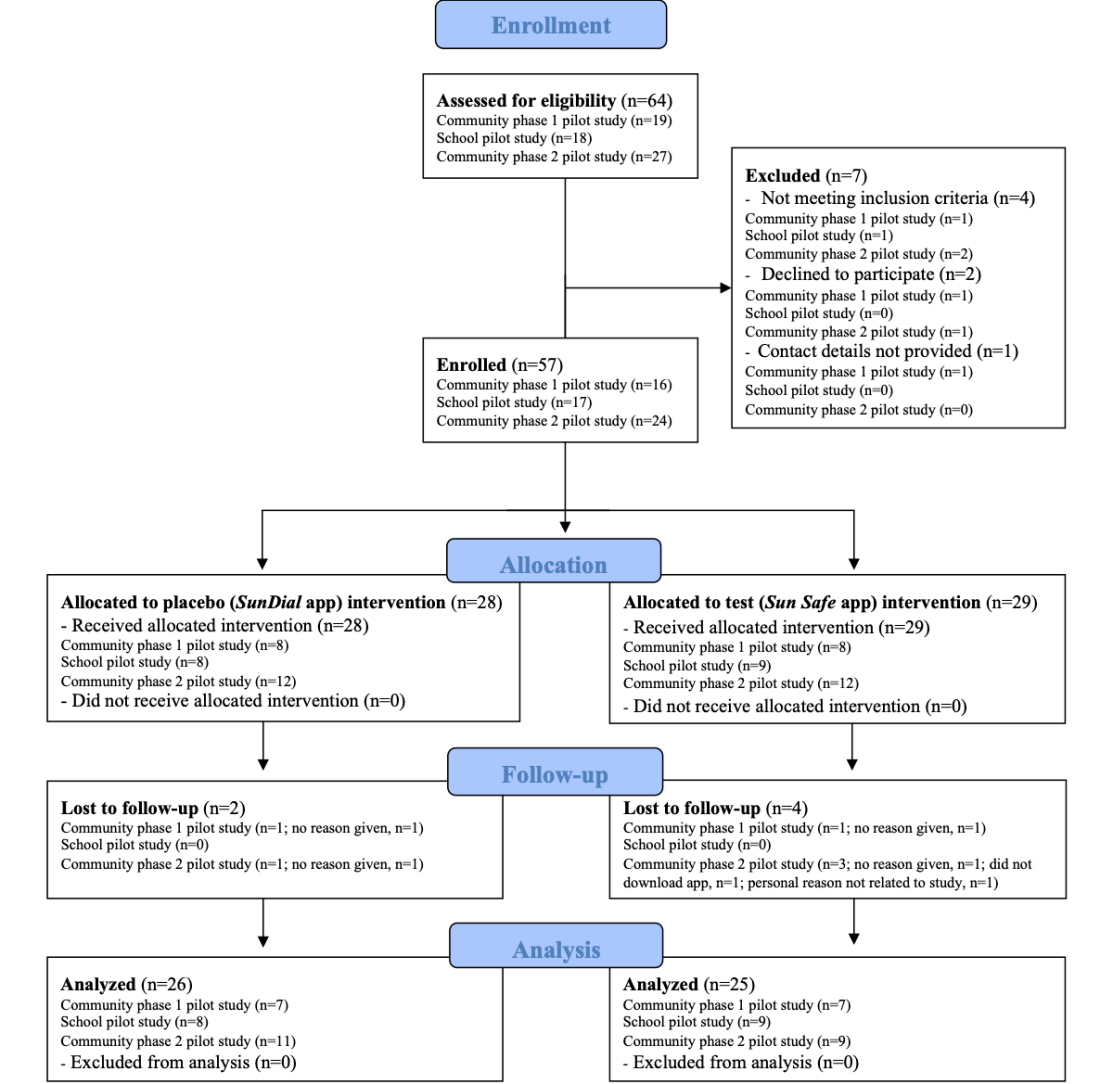
Flowchart of recruitment of participants into the 3 pilot intervention studies. For some outcomes, data were not collected for all participants or were excluded from analyses.

Eligible participants were aged 12 to 13 years and English speaking, with sufficient internet literacy to download and use the apps; had access to the internet and an Apple iOS device (ie, iPhone or iPad); and lived in WA (for community pilot studies) or attended the local school (for school pilot study). All eligible participants who provided informed consent were enrolled. A CONSORT (Consolidated Standards of Reporting Trials) flowchart detailing the enrollment of participants is shown in Figure 1.

### Study Location

**Figure 2:**
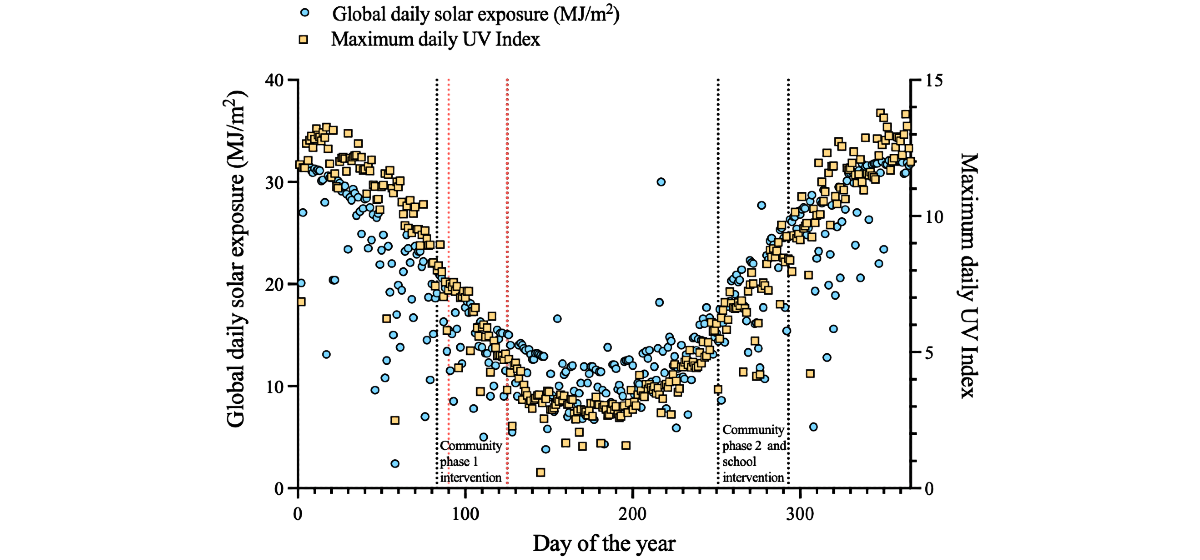
Global daily solar exposure levels and maximum daily UV Index for Perth (Western Australia) in 2020. Black broken lines encapsulate 6-week intervention periods for each pilot study. Red broken lines encapsulate the days of the year during which schools were shut due to the COVID-19 pandemic.

These studies were largely conducted in Perth, the capital city of the state of WA (latitude 31.9°S, longitude 115.9°E) [33]. The *global daily solar radiation* (total solar energy levels per day, including UV, visible, and infrared radiation) levels measured at the *Perth Metro* terrestrial weather station (Australian Government Bureau of Meteorology [34]) and maximal daily UV Index levels for Perth (Australian Radiation Protection and Nuclear Safety Agency [35]) across 2020 are shown in Figure 2. A strong and statistically significant linear correlation between global daily solar exposure levels and maximum daily UV Index was observed (Spearman test, *r*=0.84, 95% CI 0.81-0.87; *P*<.001). For more details, see Multimedia Appendix 1.

### Data Collection at Baseline

Participants were asked to provide self-assessed *baseline* responses, which were collected either through web-based questionnaires (for community pilot studies) or in-class completion of paper-based questionnaires (school pilot study). Data collected at recruitment and through questionnaires included the following:

1. Demographic information (age, gender, and postcode to estimate socioeconomic status)
2. Sun health knowledge (through completion of a multiple-choice quiz)
3. Skin type and responses to sun exposure
4. Sun health behaviors (time spent outdoors and sun-protective behaviors) and sunburn

A standardized multiple-choice quiz on sun health knowledge was developed from educational content included within the *Sun Safe* app [9] (see *Methods* section in Multimedia Appendix 1). The percentage of questions correctly answered and the time taken to complete the knowledge quizzes were recorded.

The sun health questionnaire included questions on time spent outdoors during weekdays, weekend days, and school holidays in the past 6 weeks and sun-protective behaviors at those times (wearing hats and long-sleeved or leg-covering clothing, seeking shade, and using sunscreen). Other questions included self-reported measures of sun sensitivity, tanning responses, skin type, number of moles and freckles, serious sunburns during the lifetime, and sunburns in the past 6 weeks. For more details, see Multimedia Appendix 1.

Skin type was determined by asking participants to choose a skin color they thought was closest to their own natural skin color (ie, skin of inner upper arm), which corresponded to *Fitzpatrick skin phototype color images* of types 1 to 6 (from 1=pale white skin to 6=deeply pigmented dark brown to black skin). For more details, see Multimedia Appendix 1.

In the school pilot study, self-reported sun behaviors (specifically time spent outdoors) were compared with the objective erythemally effective doses (EEDs; J/m^2^) received on school days, as measured on polysulfone dosimeters [13] worn daily by participants for 7 days immediately before and during the final 7 days of the 6-week intervention. For more details, see Multimedia Appendix 1.

### Intervention Group Allocations

**Figure 3:**
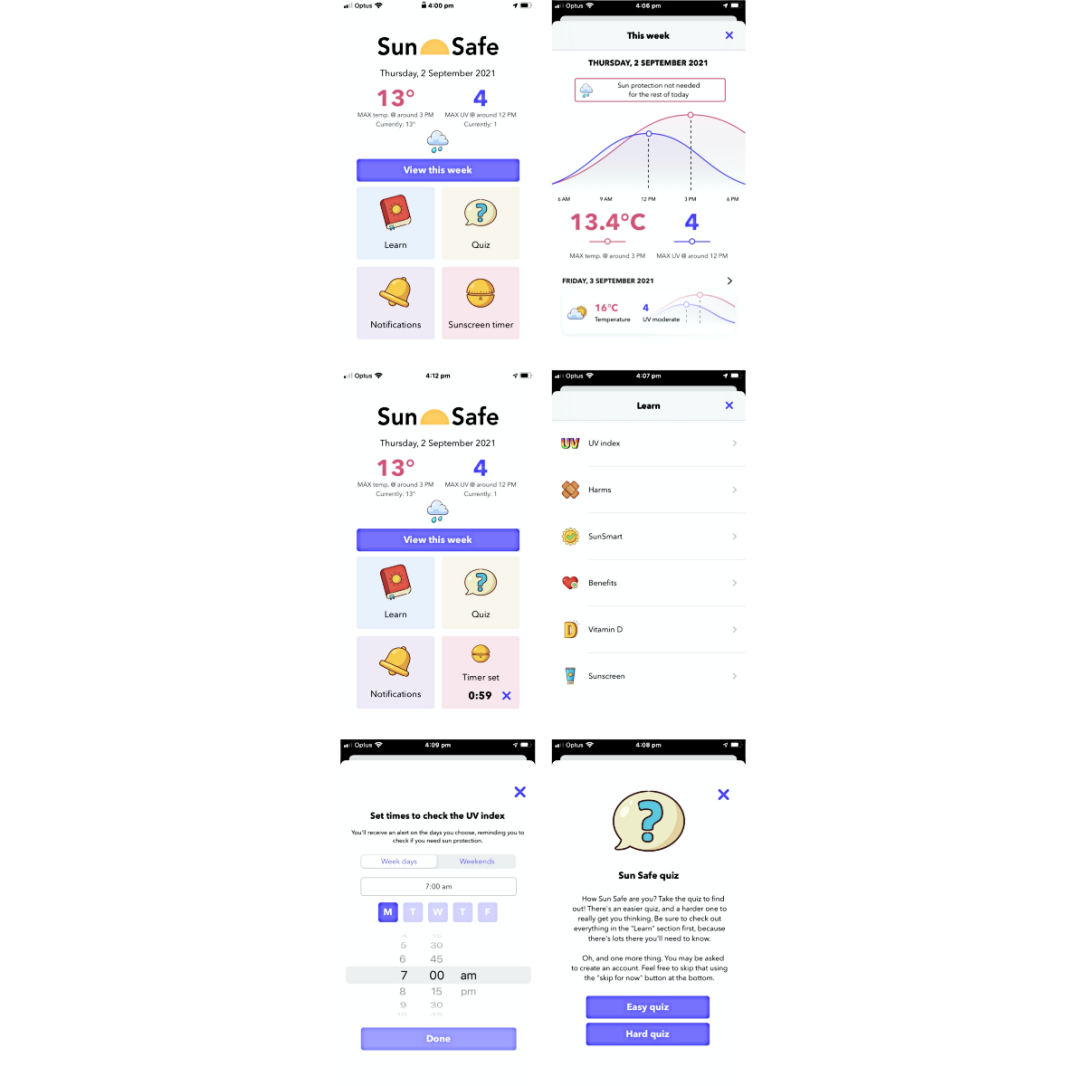
Screenshots of the Sun Safe app (clockwise from top left) include: the home page, predictive data and when to use sun protection (view this week), educational content (learn), easy and hard quizzes (quiz), notifications to check the UV Index, and a reminder to reapply sunscreen (sunscreen timer).

After the completion of baseline questionnaires, participants were allocated into 1 of 2 intervention groups, with group allocation done by matching participants (1:1) based on age, gender, and skin type. Participants were recruited through the Qualtrics platform (Experience Management; hosted at the University of WA), with enrollment and assignment of interventions managed by SG. Participants were then invited to download either the *Sun Safe* app [14] (version 1.0.1, 2020, with further development frozen during these studies; available on the Australian Apple App Store only) or a placebo app. Major features of the *Sun Safe* app are summarized in Figure 3 (see Multimedia Appendix 1 and the study by Nguyen et al [9]). The theoretical framework and co-design process underpinning the development of *Sun Safe* are reported in detail elsewhere [9]. *Sun Safe* requires the user’s location and IP address to provide location-specific information; however, these data are not stored by the app nor the provider of the information. The placebo app selected was the *SunDial* iOS app (version 6.2, 2020), which notifies the user when sunrise and sunset events occur [15]. A placebo app was required to control for the *digital placebo effect*, which may occur when being involved in a digital intervention study [16]. Participants were blinded to which were the test (*Sun Safe*) and placebo (*SunDial*) apps and were initially encouraged to download and use either app (for free) through email or information provided during an in-class session. Researchers had no further contact with the participants during the 6-week app exposure period (Figure 2).

### Data Collection After the Intervention

Data collected after 6 weeks of exposure to either app included the following:

1. Sun health knowledge (through the same multiple-choice quiz as the baseline)
2. Sun health behaviors (time spent outdoors and sun-protective behaviors) and sunburns received during 6 weeks of intervention
3. Assessments and ratings collected using a survey, which incorporated the user version of the Mobile App Rating Scale [17]

The user version of the Mobile App Rating Scale survey includes 26 items, rated on 5-point (Likert) scales, and asks users to rate the app they used across six *areas of assessment*: (1) engagement, (2) functionality, (3) aesthetics, (4) information, (5) subjective quality, and (6) perceived impact (on related health knowledge, attitudes, and behaviors) [17]. An *overall quality* rating was produced by calculating the mean score of the engagement, functionality, aesthetics, and information areas of assessment [18]. For more information, see Multimedia Appendix 1.

### Statistical Analyses

Results were analyzed using Microsoft Excel (version 16.52 for Mac, 2021) and GraphPad Prism (version 9.2.0 for Mac, 2021). Descriptive statistics were calculated, with mean and SD reported for continuous data and number and percentage (for data combined across the 3 pilot studies) for categorical data. We did not impute missing values for participants who did not complete the study, with most analyses considering data collected at baseline separate from that collected after the intervention. All data were subjected to normality tests (Shapiro–Wilk) to determine whether parametric data analyses were appropriate. Results were considered statistically significant for *P* values <.05. Unless otherwise stated, data were combined for the 3 pilot studies. For categorical data, Fisher exact tests or chi-square tests were performed to compare between intervention groups (ie, the app tested) for data combined for the 3 pilot studies. For continuous data, 2-way ANOVA with Tukey post hoc test or Student *t* test (if normally distributed) or Kruskal-Wallis test with Dunn post hoc or Mann–Whitney test (if not normally distributed) were used to determine the differences between intervention groups when data were combined across all 3 pilot studies or within each pilot study, respectively. Outcomes of the 2-way ANOVA are reported below as differences in predicted means with 95% CIs. Relative risk (RR) CIs were calculated using the Koopman asymptomatic score method. For dosimetry data, the strength of linear correlations was tested using the Pearson test. For more information, see also Multimedia Appendix 1.

## Results

### Participant Demographics

Across all 3 pilot studies, 57 participants were recruited who were given access to the placebo (28, 49% for *SunDial* [15]) and test (29, 51% for *Sun Safe*) apps (Figure 1) after matching for age, gender, and skin type, with 51 (89%) participants (26, 51% in the placebo arm and 25, 49% in the test arm) completing the studies. Overall, more participants were women who lived in postcodes of higher socioeconomic status (Socio-Economic Indexes for Areas Index of Relative Socioeconomic Advantage and Disadvantage quintiles 4 and 5) with *lighter* skin types (ie, Fitzpatrick skin types 1-3; Table 1). Approximately all individuals (56/57, 98%) lived in postcodes within the Perth metropolitan region. No statistically significant differences in gender (*P*=.99; Fisher exact test), age (*P*=.89; 2-way ANOVA), postcode-based socioeconomic status (*P*=.48; chi-square test), or skin type (*P*=.99; Fisher exact test) were observed between intervention groups (Table 1).

**Table 1:** Demographics of participants in either placebo (SunDial app) or test (Sun Safe app) intervention arms (N=57).

Demographics			Pilot study and intervention groups														
			Community phase 1				Community phase 2				School				Combined^a^		
			Placebo		Test		Placebo		Test		Placebo		Test^b^		Placebo		Test
Participants completing baseline, n^c^ (%)			8 (14)		8 (14)		12 (21)		12 (21)		8 (14)		9 (16)		28 (49)		29 (51)
**Gender, n (%)**																	
	Male	2 (25)		3 (38)		4 (33)		3 (25)		2 (25)		3 (33)		8 (29)		9 (31)	
	Female	6 (75)		5 (62)		8 (67)		9 (75)		6 (75)		6 (67)		20 (71)		20 (69)	
	Other or not stated	0 (0)		0 (0)		0 (0)		0 (0)		0 (0)		0 (0)		0 (0)		0 (0)	
Age (years), mean (SD)			12.8 (0.5)		12.9 (0.4)		12.7 (0.5)		12.8 (0.5)		12.7 (0.3)		12.6 (0.3)		12.7 (0.4)		12.8 (0.3)
**Postcode-based SEIFA^d^ IRSAD^e^, n (%)**																	
	Quintile 1	0 (0)		0 (0)		1 (8)		1 (8)		0 (0)		0 (0)		1 (4)		1 (3)	
	Quintile 2	0 (0)		2 (25)		4 (33)		2 (17)		0 (0)		1 (11)		4 (14)		5 (17)	
	Quintile 3	1 (12)		3 (38)		1 (8)		4 (33)		2 (25)		1 (11)		4 (14)		8 (28)	
	Quintile 4	0 (0)		0 (0)		3 (25)		3 (25)		3 (38)		3 (33)		6 (21)		6 (21)	
	Quintile 5	7 (88)		3 (38)		3 (25)		2 (17)		3 (38)		4 (44)		13 (46)		9 (31)	
**Fitzpatrick skin type, n (%)**																	
	1	1 (12)		2 (25)		2 (17)		2 (17)		1 (12)		0 (0)		4 (14)		4 (14)	
	2	3 (38)		4 (50)		5 (42)		5 (42)		1 (12)		1 (11)		9 (32)		10 (34)	
	3	4 (50)		2 (25)		3 (25)		3 (25)		3 (38)		6 (67)		10 (36)		11 (38)	
	4	0 (0)		0 (0)		2 (17)		2 (17)		1 (12)		1 (11)		3 (11)		3 (10)	
	5	0 (0)		0 (0)		0 (0)		0 (0)		2 (25)		1 (11)		2 (7)		1 (3)	
	6	0 (0)		0 (0)		0 (0)		0 (0)		0 (0)		0 (0)		0 (0)		0 (0)	

^a^For data combined across the 3 pilot studies, statistical comparisons were made between placebo and test interventions for the following: gender: RR=0.9 (95% CI 0.4-2.0); *P*=.99; Fisher exact test; age: –0.02 years difference in predicted means (95% CI –0.24 to 0.28); *P*=.89; 2-way ANOVA with Tukey post hoc test; SEIFA Index of Relative Socioeconomic Advantage and Disadvantage: *P*=.48; chi-square test; groups collapsed as described in the *Methods* section; Skin type: RR=0.9 (95% CI 0.5-1.6); *P*=.99; Fisher exact test; groups collapsed as described in the *Methods* section.

^b^One test participant did not complete the baseline surveys as they were not able to attend the in-school session.

^c^Participants recruited into each pilot study who completed all baseline questionnaires and were given access to either the placebo (*SunDial*) or test (*Sun Safe*) apps for 6 weeks.

^d^SEIFA: Socio-Economic Indexes for Areas.

^e^IRSAD: Index of Relative Socioeconomic Advantage and Disadvantage.

### Skin Sensitivity, Tanning Responses, and Number of Moles and Freckles

At baseline, there were no statistically significant differences in skin-burning (sensitivity) or tanning responses to 30 minutes of exposure to summer sunlight, skin appearance at the end of summer, or number of moles or freckles between the test (*Sun Safe*) and placebo groups (Multimedia Appendix 1 Table S1).

### Downloading and Using the Apps

In the community pilot studies, there were no significant differences in the time taken to download the apps (*P*=.64; Mann–Whitney test) or time for which apps were accessed (*P*=.20) between the placebo and test (*Sun Safe*) groups (Multimedia Appendix 1 Table S2). Most participants used a smartphone (>50%) to access their designated app either once a fortnight or once or twice (in total).

### Sun Health Knowledge Was Increased With Exposure to the Sun Safe App

**Figure 4:**
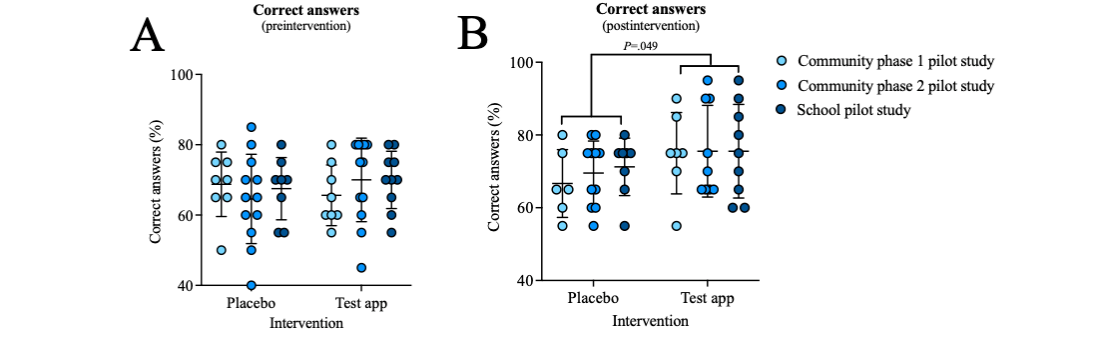
Exposure to the test app (Sun Safe) increased the percentage of questions correctly answered by participants (in a 20-question multiple-choice quiz) across all 3 pilot studies. Data collected during (A) preintervention assessment (28/28, 100% placebo and 29/29, 100% test) and (B) postintervention assessment (25/28, 89% placebo and 25/29, 86% test) were compared using 2-way analysis of variance (with Tukey post hoc analysis; −6.2% difference in predicted means, 95% CI –12.4 to –0.03; *P*=.049, 2-way analysis of variance). One participant from the placebo arm of the school pilot study did not attend the in-school session during which the multiple-choice quiz was conducted at the postintervention time point. Data are shown as mean (SD).

Participants completed a 20-question multiple-choice quiz before (Figure 4A) and after (Figure 4B) the 6-week intervention. Participants who were given access to the *Sun Safe* (test) app demonstrated greater sun health knowledge than those in the placebo group (Figure 4B; −6.2%, 95% CI –12.4% to –0.03%; *P*=.049, 2-way ANOVA). Specific knowledge improvements were about the UV Index, with significantly more participants from the *Sun Safe* group correctly answering the question, “At which UV Index values are sun protection recommended when you are outside?” (ie, 13/25, 52% in placebo and 20/25, 80% in test arms answered correctly; RR=0.65, 95% CI 0.41-0.97; *P*=.04; chi-square test; Multimedia Appendix 1, Table S3). There was no difference between men and women in the percentage of correct answers achieved before or after the intervention (Multimedia Appendix 1).

### Sunburns

There were no statistically significant differences in the number of serious sunburn events reported across the lifetime or any sunburn during the 6 weeks before the intervention between the groups (Table 2). However, there were significantly more sunburn events reported by participants in the *Sun Safe* group during the 6 weeks of the intervention than those in the placebo group (Table 2; RR=1.7, 95% CI 1.1-2.8; *P*=.02; Fisher exact test). Within the *Sun Safe* group, these were mostly (10/13, 77%) not *bad sunburns*. No statistically significant difference observed between groups in the number of bad sunburns (RR=0.5, 95% CI 0.1-1.2; *P*=.27; Fisher exact test).

**Table 2:** Sunburns during lifetime or the 6 weeks before or during the intervention^a,b^.

Intervention group		Before intervention (combined; n=56), n (%)			During intervention (combined; n=51), n (%)			
		Placebo	Test	Placebo			Test	
Participants		28 (50)	28 (50)	26 (51)			25 (49)	
**Lifetime sunburns^c^**						N/A^d^		N/A
	0	7 (25)	7 (25)					
	1	4 (14)	7 (25)					
	2-10	11 (39)	11 (39)					
	>10	4 (14)	1 (4)					
	Do not know	2 (7)	2 (7)					
**Frequency of sunburn in the past 6 weeks**								
	Never	19 (68)	21 (75)	21 (81)			12 (48)	
	Once	7 (25)	4 (14)	3 (12)			10 (40)	
	2-10 times	1 (4)	2 (7)	1 (4)			2 (8)	
	>10 times	0 (0)	0 (0)	1 (4)			0 (0)	
	Do not know	1 (4)	1 (4)	0 (0)			1 (4)	
**How many of these were bad sunburns?^e^**								
	0	5 (62)^f^	4 (67)^g^	2 (40)^h^			10 (77)^i^	
	1	3 (38)^f^	1 (17)^g^	2 (40)^h^			2 (15)^i^	
	2-10	0 (0)^f^	1 (17)^g^	1 (20)^h^			1 (8)^i^	
	Do not know	0 (0)^f^	0 (0)^g^	0 (0)^h^			0 (0)^i^	

^a^Data are shown as number (n) of each participant who selected each response and percentage within each intervention group, with data combined from participants enrolled in 1 of 3 pilot studies, who completed the survey before and after 6 weeks of access to either the placebo (*SunDial)* or test (*Sun Safe*) apps.

^b^Statistical comparisons were made between placebo and test interventions using the Fisher exact test (with groups collapsed, as described in *Methods* section of Multimedia Appendix 1) for the following: lifetime sunburn: RR=0.8 (95% CI 0.4-1.4); *P*=.59; frequency of sunburn (before): RR=0.9 (95% CI 0.6-1.3); *P*=.77; Frequency of sunburn (during): RR=1.7 (95% CI 1.1-2.8); *P*=.02; bad sunburns (during): RR=0.5 (95% CI 0.1-1.2); *P*=.27.

^c^Number of sunburns to a significant area of skin with pain lasting longer than a day, experienced in a lifetime (asked only at baseline; ie, before intervention).

^d^N/A: not applicable (as data were only collected at baseline).

^e^For those who experienced any sunburn in the past 6 weeks, how many of these were bad sunburns to a significant area of skin, with pain lasting longer than a day?

^f^n=8.

^g^n=6.

^h^n=5.

^i^n=13.

### Time Spent Outdoors

There were no statistically significant differences in the time spent outdoors either before or during the intervention period between the placebo and test groups (Multimedia Appendix 1 Table S4). There were also no statistically significant differences in the time spent outdoors between the placebo and test groups either before or during the intervention within each pilot study (Multimedia Appendix 1 Table S4).

**Figure 5:**
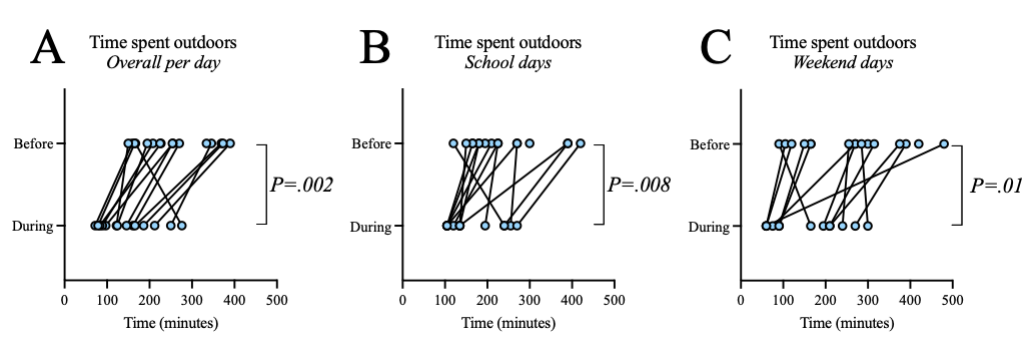
Time spent outdoors was significantly reduced during the intervention period for participants of the community phase 1 pilot study. Data collected before (16/16, 100%) and during the intervention (13/16, 81%) were compared using paired Student t tests (*P*<.05), including (A) overall time spent outdoors per day (−105 minutes difference in predicted means, 95% CI −150 to −59 minutes; *P*=.002), (B) time spent outdoors on school days (−81 minutes, 95% CI −135 to −26 minutes; *P*=.008), and (C) time spent outdoors on weekend days (−96 minutes, 95% CI −169 to −23 minutes; *P*=.01). Data are shown for each individual and paired for responses before and during the intervention period (combined for both intervention groups).

Within the community phase 1 pilot study, significant reductions in time spent outdoors were observed during the intervention compared with the time before the intervention (Figure 5A-5C; overall: –105 minutes, 95% CI –150 to –59 minutes; *P*=.002; school weekdays: –81 minutes, 95% CI –135 to –26 minutes; *P*=.008; weekend days: –96 minutes, 95% CI –169 to –23 minutes; *P*=.01, paired Student *t* test). This was notable, as the intervention ran across the initial COVID-19 pandemic–induced shutdown period of April 2020. Significant reductions in time spent outdoors occurred in the late afternoon (3 PM to 6 PM) on school days (before: mean 75, SD 40 minutes; during: mean 40, SD 33 minutes; *P*=.03; Wilcoxon test) and in the middle of the day (10 AM to 2 PM) on weekend days (before: mean 81, SD 47 minutes; during: mean 53, SD 39 minutes; *P*=.049; paired Student *t* test). These observations were not reproduced in the other pilot studies (Multimedia Appendix 1 Table S4 and data not shown, respectively) suggesting that the reduction in time spent outdoors was an effect of the COVID-19 pandemic.

### Validation of Time Spent Outdoors With Dosimetry Data

Overall, the number of EED received by participants increased as time spent outdoors on school days increased, with a significant positive linear correlation observed before the intervention (Multimedia Appendix 1 Figure S1; Pearson *r*=0.67, 95% CI 0.22-0.89; *P*=.008). For more data related to wearing dosimeters, including compliance, please see Multimedia Appendix 1 Figure S1 and Table S5.

### Personalized UV Exposure Measured by Dosimeters in School Pilot Study

There was no difference between UV exposure levels (ie, EED) measured via dosimeters worn by school pilot study participants in the placebo and test groups in the week before or last week of the intervention (Multimedia Appendix 1 Figure S1).

### Sunscreen Use and Sun-Protective Behaviors

The preferred mode of sun protection by participants was seeking shade (Multimedia Appendix 1, Tables S6 and S7). No significant differences in the use of sunscreen were observed before or during the intervention between the placebo and test groups (Multimedia Appendix 1 Table S6). There was little difference in other sun-protective behaviors (including seeking shade, wearing a hat, or wearing clothing with long sleeves) on school days (between 10 AM and 3 PM; Multimedia Appendix 1, Table S7) and weekend days (between 10 AM and 2 PM; data not shown).

### Sun Safe Was Rated Higher Across Most Areas of Assessment

When data were combined across all pilot studies, *Sun Safe* was rated highest for information (mean 4.2, SD 0.6) and lowest for engagement (mean 2.9, SD 0.6; Multimedia Appendix 1, Table S8). Across all areas of assessment except aesthetics, *Sun Safe* was rated significantly higher than the placebo app (Multimedia Appendix 1, Table S8; for combined data). Participants using *Sun Safe* were more likely to recommend it to others (*P*=.003; Mann–Whitney test) and use it more frequently in the next 12 months (*P*=.008) than those using the placebo app (Multimedia Appendix 1 Table S9). Only 12% (3/24) of the participants stated that they would pay for the *Sun Safe* app (Multimedia Appendix 1, Table S9).

## Discussion

### Principal Findings

Here, we describe how exposure to the *Sun Safe* app increased the knowledge that young Australian teenagers living in Perth (WA) had about the UV Index through placebo-controlled pilot intervention studies. Participants exposed to *Sun Safe* rated it highly, particularly for *information*. With some emphasis on the benefits of sun exposure, we may have expected that *Sun Safe* would increase the time spent outdoors using sun protection. However, no differences were observed in the time spent outdoors or sun-protective behaviors. These behaviors were likely strongly influenced by the COVID-19 pandemic. Indeed, during the shutdown period of April 2020, there was significantly reduced time spent outdoors observed in participants of the community phase 1 pilot study (mean 105, SD 78 minutes per day). This was likely linked to reduced opportunities to participate in outdoor sporting activities and the capacity of participants to engage in extracurricular outdoor activities. A participant stated that there was “no organized sport due to the COVID-19 pandemic.” Others have also reported reduced time spent outdoors by children living in Israel during COVID-19 restrictions [36]. There was increased reporting of (not bad) sunburns during the intervention period in the *Sun Safe* group compared with the placebo group. As there was no difference in time spent outdoors or reported sun behaviors between interventions, it may be that this increase in sunburns was because of increased awareness of the impacts of skin exposure to excessive sunlight, so that users of *Sun Safe* were more aware of sunburns and therefore more likely to recognize and report them.

Although *Sun Safe* described some benefits of sun exposure, *using sun protection as indicated by the UV Index* was prioritized within the *learn* feature and across all app features (eg, *View this week* for when to use sun protection and *Quiz* questions [9]). Information on *harms* and *SunSmart* behaviors featured first in the *learn* feature. However, it is possible that sun behaviors worsened with exposure to *Sun Safe*, with these pilot studies insufficiently powered to detect significant changes in behavior. Indeed, a systematic review recently identified unexpected consequences of using the UV Index to make health decisions, such as intentional tanning [37]. It may be that using the UV Index to make sun health decisions is not the best approach for young teenagers, and sun health apps that target this age group need to promote sun-protective behaviors more generally. However, it is important to recognize the small sample size (N=57) of these pilot studies and that further studies are required with larger cohorts to reproduce and better understand these findings.

Using *Sun Safe* significantly increased important sun health–related knowledge among young teenagers, with no differences observed between male and female participants. This was perhaps unexpected as we observed less engagement of male coresearchers during the co-design process, with fewer men than women recruited as coresearchers, and some uncertainty regarding how feedback from male coresearchers translated into the development of *Sun Safe* [9]. Male coresearchers also displayed a sense of indifference regarding sun protection through interviews conducted as part of the *Sun Safe* co-design process [38]. Whether these increases in sun health knowledge translate into improved sun-protective behaviors by men is uncertain. Other uncertainties exist regarding whether knowledge gains observed for *Sun Safe* will have long-term effects on behavior with a relatively short intervention period (6 weeks) tested here.

A strength of these pilot studies was the relatively low dropout rate (approximately 10% overall) compared with the findings of a systematic review of intervention studies that included intervention lengths that ranged from 10 days to 6 months and tested mental health apps for which much higher (>25%) losses to follow-up were observed [19]. Another strength was the use of the *SunDial* app to control for the *digital placebo effect*, which may come about in digital intervention studies through positive expectations of receiving beneficial effects, as personal devices such as smartphones may be an *extension of self* [16]. The inclusion of digital controls may be essential to determine real-world effectiveness, with many mental health apps not demonstrating therapeutic effectiveness when a digital control was included as a comparator group [39,40]. *SunDial* was chosen as, although its focus was on the sun, no information related to sun health was imparted. It was free to download, included no in-app advertisements, and had few privacy concerns.

Blinding users to placebo and test apps is an ongoing challenge in digital health intervention studies. To aid this process, we included knowledge quiz questions related to the nature of the placebo app, which notify the user when sunrise and sunset events occur. However, it is uncertain whether *SunDial* was the best placebo app to use. A modified or disabled version of *Sun Safe* could be used as a placebo, although this might be obvious to participants (depending on the modifications made) and was beyond our funding budget. Furthermore, it is difficult to determine which features would be best excluded as the effective components of *Sun Safe*. Another approach could be to have a *no app* control group; however, this would not adequately control for the *digital placebo effect* [16]. Including a third, *no app* control group could be considered, as well as different experimental approaches, such as incorporating a crossover design (although this still might not overcome issues regarding blinding) or by testing another health app in a side-by-side fashion and including questions in surveys (or other) that also measure the health outcomes of the alternate app.

### Limitations

Limitations of these pilot studies include biases in participant recruitment, particularly for gender, socioeconomic status, and skin type. Most participants were recruited from the Perth metropolitan area, and thus, it is unclear whether the methods used, and the findings of these pilot studies are applicable elsewhere. Future intervention studies should aim to increase the diversity of participants recruited (considering gender, socioeconomic status, skin type, and residence beyond metropolitan Perth). These could use a combined web-based and school recruitment strategy (managed via the web), targeting schools attended by students living in more disadvantaged Socio-Economic Indexes for Areas to increase participant numbers and diversity. Recruitment media and communications could also be provided in languages other than English for the recruitment of young people from culturally and linguistically diverse backgrounds. Further development of *Sun Safe* may be necessary to improve accessibility (ie, an Android version and language options) and engagement, which might be addressed by additional gamification suggestions raised by coresearchers during the *Sun Safe* co-design process (ie, incorporation of in-app minigames [9]). Other researchers have recently developed potentially engaging virtual reality games that promote sun protection [41]. The information content of *Sun Safe* may also need to be modified, particularly if an increased risk of sunburn persists in future (better powered) studies. Factors that may have affected recruitment in our pilot studies, which may be hard to address in future studies, could include parental concerns over smartphone use and the web-based environment, potential resistance by some young people to participate if recruited through their parents, and the ongoing influence of the COVID-19 pandemic. We now have a better understanding of the sample size requirements of future intervention studies, with sufficient sample size (N=57) demonstrated for user knowledge improvements but perhaps not for differences in sun-protective behaviors. Other limitations include those typical of eHealth trials, such as nonblinding of participants, the number of outcomes assessed (and risk of type 1 error), and biases introduced by limited use of the apps tested.

### Conclusions

Skin cancers are the most prevalent form of cancer (affecting 2 in 3 adults) in Australia and bring substantial health and economic costs (eg, >Aus $1 billion [US $0.7 billion] in 2015-2016 nationally [42]), with prevention 30-fold less costly than treatment [43]. Adolescents are a key target population for skin cancer prevention campaigns and education, through which relatively small investments could bring about significant health and cost savings. Some sun exposure is important for maintaining vitamin D levels as teenagers become young adults, a population at risk for vitamin D deficiency in Australia [44]. We demonstrated that the use of the *Sun Safe* app in real-world settings improved the sun health knowledge that young teenagers have about the UV Index. Larger intervention studies in community and school settings with greater statistical power are needed to reproduce these findings and determine whether this app affects sun health behaviors.

## Appendix

Multimedia Appendix 1 Additional methods and results.

## Abbreviations

CONSORT: Consolidated Standards of Reporting Trials
CONSORT-EHEALTH: Consolidated Standards of Reporting Trials of Electronic and Mobile Health Applications and Online Telehealth
EED: erythemally effective dose
RR: relative risk
WA: Western Australia
