# Supplementary material for: The Effects of Using the Sun Safe App on Sun Health Knowledge and Behaviors of Young Teenagers: Results of Pilot Intervention Studies
Source: JMIR Dermatol. 2022 Mar 16;5(1):e35137. doi: 10.2196/35137 (PMC10334904; doi:10.2196/35137)
Supplement: Multimedia Appendix 1 [file derma_v5i1e35137_app1.pdf]

Multimedia Appendix

Title:
Use of the *Sun Safe* app by young teenagers increased their knowledge of the UV Index: results of pilot intervention studies.

Authors:
Isabelle M Clare, Nisali Gamage, Gail A Alvares, Lucinda J Black, Jacinta Francis, Robyn M Lucas, Mohinder Jaimangal, Mark Strickland, James White, Rebecca Nguyen and Shelley Gorman

Methods

Informed consent
To obtain informed consent the following steps were conducted:

i) Participants were invited to enrol in the study through an online portal (hosted on the Telethon Kids Institute website).

ii) Participants and parents were directed to an online consent form (hosted on Qualtrics), through which they acknowledged that they had read the Participant or Parent Information Sheets (respectively) and understood what was involved in participating in the study.

iii) Participants and parents then agreed to participate in the study and to provide their name, gender (participant only) and postcode of residence.

In the School pilot, informed consent was first sought from the school to participate in the study via a letter and information and consent form sent to the school principal. For community pilots, participants and parents were also asked to provide their email address and contact numbers. Information sheets provided to participants, parents and the school included details such as: the aim of the study; possible benefits; requirements of participation; how long each survey was anticipated to take to complete; which data would be stored and for how long; and the name and contact details of the lead investigator.

Recruitment of participants
Recruitment notices placed on the Telethon Kids Institute's Facebook page were shared with community groups (e.g., health promotion, local government, parenting, cyber-safety, nature and science, and sporting groups; n=158 organisations) via Facebook, email and/or community-based seminars. The Telethon Kids Institute logo was displayed on all recruitment media and documents. Wording of the social media notices was: "we're looking for 12 and 13-year-olds to help us test a new app designed to promote healthy sun behaviours in young people. If you think your child would be interested in taking part in this real-life research, we'd love to hear from you!" Further links to the study website were provided where participants could access more information on eligibility and requirements of participants, name of lead investigator and contact details, and information and consent forms. In the School pilot, participants were contacted through in-class sessions organised with the school and participating teachers, with a presentation given by a researcher, and potential participants given a flyer which stated: "We need your help to test a new health app designed to promote healthy behaviours." Further links to the study website were provided where participants could access more information on the eligibility and requirements of participants, name of lead investigator and contact details, and information and consent forms.

COVID-19 pandemic impacts on recruitment and timelines of pilot intervention studies

Recruitment to perform pilot intervention studies began on 11 February 2020 for the Community phase 1 and School pilots. From the 16 March 2020, baseline responses were collected either through online questionnaires (for the Community phase 1 study) or in-class completion of paper questionnaires (School pilot). On the 23 March 2020, the School pilot was suspended because of ongoing issues around containing spread of COVID-19 with concerns that continuing to conduct in-class sessions would be too risky for participants, researchers, and the wider school community. 'Non-essential venue shutdowns' were being recommended around Australia from noon that day. WA schools were closed to most students from the 30 March until the 4 May 2020. As shown in Figure 2, the broken black lines at the 83rd (March 23, 2020) and 125th (May 4, 2020) days encapsulate the 6-week intervention period for the community phase 1 pilot study. The broken black lines at 251st (September 7, 2020) and 293rd (October 19, 2020) days encapsulate the 6-week intervention period for community phase 2 and school pilot studies. The red, broken lines correspond to the days of the year (ie, days 90-125; from March 30, 2020, to May 4, 2020) during which schools were shut for most students owing to restrictions imposed as part of attempts to contain the COVID-19 pandemic.

The Community phase 1 pilot was continued across this shutdown period with minor modifications made to questionnaires to allow participants to provide answers related to being home-schooled. On the 23 March 2020, participants were invited to download either the *Sun Safe* or placebo app, with post-intervention data collected from the 4 May (via online questionnaires). As researchers considered it highly likely that the capacity of participants to spend time outdoors would be changed by the impacts of the April 2020 shutdown period, a second community-based study started recruiting participants on the 27 July 2020 (Community phase 2 study). The School pilot also recommenced on the 27 July 2020. For these two pilots, on the 7 September 2020 participants were allocated (matching for age, gender, and skin type) into the *Sun Safe* or placebo app intervention groups and invited to download their respective apps, with post-intervention data collected from the 19 October 2020 for these two pilots.

Study location

Average daily levels of sunshine in Perth annually are 8 hours [20]. Perth is a coastal city of 2.1 million people [21] located ~10 km from the Indian Ocean, adjacent to the Swan River, with a warm climate throughout much of the year [22].

Survey development and administration

Information provided here and in the main text conforms with the Checklist for Reporting Results of Internet e-Surveys (CHERRIES). All surveys (both online and paper) were tested by investigators, consumer advocates, officials from the Department of Education of Western Australia, and a separate group of young teenaged consumers for age-appropriate use of language and technical functioning. Participants were provided specific links to web-based surveys (hosted on the Qualtrics platform) via email or paper versions of these surveys in-class. Survey items were not randomised. Adaptive questioning was applied specifically for collection of sun behaviour data. Depending on the length and complexity of questions posed, up to 7 questions per page were posed for online surveys with up to 35 screens/pages, except for the sun health knowledge quiz for which all questions were posed on a single page. Warnings were included to prompt responses if incomplete, with options to select 'not applicable' or 'rather not say' if appropriate. Prior to submission of surveys, a 'back' button enabled participants to review answers. Participants were asked to provide their name to identify who completed online surveys, and their status as a unique site visitor determined via collection of a unique IP address with no duplicate entries identified. Participants were paid honorarium of Aus $25 (US $18) upon completing all surveys.

Data protection and risk management

Encrypted recruitment data and other online survey data was stored on the Qualtrics platform. Once pilot intervention studies were completed, data were immediately downloaded, deidentified and securely stored at Telethon Kids Institute. All paper-based data was stored in locked filing cabinets, in a secure location at the Telethon Kids Institute. Electronic data were stored on password-protected computers in a secure location accessed only by investigators who had signed confidentiality agreements. The means to re-identify data was kept in a separate location to original data files. Privacy of participants' information was assured through linkage to a study identification number for data de-identification, with limited collection of personal data (name, age, gender, postcode). Privacy policies of the developed apps were provided during the recruitment process. We did not foresee any major risks associated with participating in this research project, although we did ask participants to inform parents of any sunburn events and seek medical attention if treatment was required during the trial.

Standardized multiple-choice quiz on sun health knowledge

The time taken to complete the 20-question quiz was recorded in minutes and seconds. Participants were given the following advice: that this quiz would likely take no longer than 20 min to complete; to choose the answer which was most correct; and, that some questions may not be relevant to the app they were given access. These questions were included as a diversion from the intent of the trial and instead focused on the intent of the placebo app (i.e., times of sunrise and sunset). The specific questions posed to participants, and answer choices are shown below with correct answers underlined (removed prior to administration of quizzes).

1. Sunburn increases the risk of which cancer?
   a. Stomach cancer
   b. Brain cancer
   c. <u>Skin cancer</u>
   d. Leukemia

2. Which wavelengths of sunlight are most risky for sunburn, and for making beneficial vitamin D in your skin?
   a. Infra-red radiation
   b. Ultraviolet A radiation
   c. <u>Ultraviolet B radiation</u>
   d. Visible light

3. True or False? Solstice events occur in autumn and spring.
   a. True
   b. <u>False</u>

4. At which UV index values are sun protection recommended when you are outside?
   a. 1 and above
   b. 2 and above
   c. <u>3 and above</u>
   d. None of the above

5. Which cancer is the most common cancer in young adults in Australia?
   a. Leukemia
   b. <u>Melanoma</u>
   c. Brain cancer
   d. Thyroid cancer

6. The equinox is the time(s) or date(s) at which the sun crosses the celestial equator. How many times a year does this occur?
   a. Once
   b. <u>Twice</u>

   c. 12 times
   d. Weekly

7. What is the best definition of the UV index?
   a. The UV index is forecast every day by the Bureau of Meteorology
   b. The UV index describes the amount of UV radiation at any time of day
   c. The UV index can be used to help determine when sun protection is needed
   d. <u>All of the above</u>
   e. None of the above

8. Which of these factors does NOT affect the UV index?
   a. Time of day
   b. Season
   c. Cloud cover
   d. How close you are to the Equator
   e. <u>Wind speed</u>

9. True or False: There are other benefits of sun exposure than vitamin D.
   a. <u>True</u>
   b. False

10. Too much sun exposure may cause:
   a. Skin cancer
   b. Eye damage
   c. Wrinkles
   d. <u>All of the above</u>
   e. None of the above

11. True or False: People with fair skin are at increased risk of skin cancer compared to those with dark skin.
   a. <u>True</u>
   b. False

12. When you need sun protection outside, you should:
   a. Put on a hat
   b. Seek shade
   c. Wear long-sleeved clothing to cover skin
   d. Wear sunglasses
   e. Apply sunscreen
   f. <u>All of the above</u>
   g. None of the above

13. Does shade provide complete protection from UV radiation?
   a. Yes (full shade provides 100% protection)
   b. <u>No (shade provides some protection, but you should also consider reflected UV radiation)</u>

14. Where does Western Australia rank in terms of rates of skin cancer among Australian states and territories?
   a. 1st
   b. <u>2nd</u>
   c. 3rd
   d. 4th

15. True or False: Oily fish are a good dietary source of vitamin D.
   a. <u>True</u>
   b. False

16. When you need sun protection outside, broad-spectrum 30+ SPF sunscreen should be applied at least:
   a. <u>Every 2 hours</u>
   b. Every 4 hours
   c. Every 6 hours
   d. Not at all

17. When does the vernal equinox occur?
   a. Summer
   b. Spring
   c. Autumn
   d. Winter

18. True or false: You can make vitamin D while driving in the car if your skin is exposed to sunlight coming through the window glass.
   a. True
   b. False

19. Select the missing words.
It is necessary to expose __________ *to sunlight to produce vitamin D.*
   a. Your hair
   b. Your skin
   c. Your nails
   d. Your clothes

20. True or False: Tanning increases your risk of skin cancer.
   a. True
   b. False

Sun questionnaire

Questions related to determining UV exposure, particularly time spent outdoors were validated previously in the *45 and Up Study* using UVB dosimetry [23] and have been used in other sun health intervention trials [24].

Skin type

Self-report of natural skin colour was appropriate to use in these studies in which we were not able to use spectrophotometry and were similar to visual scales shown to be superior to questionnaires for skin phototype self-assessment by children in other studies [25]. In addition, self-report of natural skin colour previously significantly correlated with Fitzpatrick skin type when determined by questionnaire (r=0.63, *P*<.001) [26].

UV dosimeters in the School pilot

Participants were instructed to wear dosimeters inserted into a silicon wristband (on their non-preferred hand) every day for 7 days immediately before, and for the final 7 days of the 6-week intervention period. A new dosimeter was worn every day. Dosimeters were stored in the dark when not worn and were collected by researchers at the end of each 7-day period. Participants were instructed to complete a paper-based compliance chart to record that they had (or had not) worn the dosimeter on the required days, with space provided to record additional information in case something happened to the dosimeter (e.g., it was worn in the shower or was lost). Data were excluded for days participants indicated (through compliance chart entries) that they did not wear their badges and for badges worn on the first day as most were put on at 3 PM (during in-class sessions).

Erythemally effective doses (EED) were calculated from the polysulphone badges. The EED received are weighted on the spectral distribution of UV radiation incident to the subject with erythemal effectiveness set by the Commission Internationale de l'Eclairage across the UV radiation wavelength range (280 to 400 nm, J/m^2). EED were calculated by determining the difference in change from baseline in optical density of the polysulphone plastic of each dosimeter at wavelengths of 330 nm using the Agilent Cary 60S UV-Vis spectrophotometer, converting the $Absorbance_{330}$ (x) to 'raw EED' (y) as previously [27], using the following formula:

$$y=(-250.9)+(14890-(-250.9))/(1+(10^{((0.7673-x)*2.316)}))$$

The ‘final EED’ was determined by further subtracting the EED values for polysulphone negative controls (i.e., not exposed to sunlight) from the ‘raw EED’ values. To cross-validate polysulphone dosimetry, a researcher wore the polysulphone dosimeters with an alternate dosimeter (‘My Skin Track UV’ dosimeter, La Roche-Posay [28,29]) on the same days as participants, which calculates the percentage of UVA, UVB and ‘My UV dose’ received depending on the person’s skin type (which was Type I/II for this researcher).

Features of the *Sun Safe* app

Major features of the *Sun Safe* app are depicted in Figure 3 (see also [9]), and included:

1. A home page with that day’s maximal temperature and UV index.
2. Predictive data on the maximal temperature and UV index linked to times when to use sun protection for that day and subsequent days, accessed through *view this week* button.
3. Educational content in *learn* feature.
4. *Easy* and *hard* quizzes in *quiz* feature.
5. Capacity to set daily *notifications* to check the UV index
6. A reminder to reapply sunscreen set with a countdown, after which a notification is sent.

The placebo app

The *SunDial* app was freely available to download from the Australian Apple app store. While its focus is on the sun, no information is imparted related to sun health. Importantly, there is no in-app advertising and few privacy concerns related to using this app, which does not collect any data from the user’s device and uses GPS and built-in algorithms to calculate sunrise and sunset times for notifications.

The uMARS survey

The uMARS survey was initially developed by the Young and Well Cooperative Research Centre and the Queensland University of Technology. It is an alternative to brief and non-specific 5-star ratings available via online app stores [30]. A modified version of the uMARS survey was used (as previously [31]), supplemented with additional questions on the device used to test the app and frequency of use of each app, and open questions that asked participants to provide qualitative responses on their opinions of each area of assessment. These qualitative data and detailed analyses of findings of the uMARS survey by gender will be reported elsewhere. We asked all participants to complete the uMARS surveys so that:

- Comparative assessments could be made as to how the test app was received by young adolescents, compared to a placebo app which was not necessarily designed to target young teenagers; and,
- Participants in the placebo group had the same experience as those in the test group.

Statistical analyses

Socio-Economic Indexes for Areas (SEIFA) quintile rankings (from most disadvantaged (1) to most advantaged (5)) for each participant’s suburb of residence were determined using the Index of Relative Socio-economic Advantage and Disadvantage (IRSAD) Interactive Map on the Australian Bureau of Statistics website [32]. A paired Student’s test was used for comparisons made between data collected before and during interventions within pilot studies. A Welsh t test was used instead of Student’s t test if unequal variances were detected (using the F test). We also attempted to perform multivariate regression analyses, in which adjustments for covariates (i.e., gender, age, skin type, SEIFA, pilot study) could be made. However, these models failed due to the relatively small sample size of this study, some missing data (i.e., for EED on school days), and the number of covariates to be tested. For categorical comparisons, data were collapsed across some categories so that there were ≥5

units per category including for: SEIFA (quintiles ‘2’ and ‘3’); Fitzpatrick skin types ((‘I’ and ‘II’),(‘III’, ‘IV’ and ‘V’); skin reaction to 30 min of sunlight (‘get mildly sunburnt’, ‘have painful sunburn’, ‘get severe sunburn’, and, ‘uncertain’); get a tan (‘no’ and ‘don’t know’); skin appearance at end of summer ((‘no suntan’ and ‘lightly tanned’), (‘moderately’ and ‘very tanned’)); moles (‘a few’, ‘some’ and ‘many moles’); freckles (‘a few’, ‘some’ and ‘many freckles’); lifetime sunburn events (‘none’ and ‘one’); lifetime sunburn events (‘2 to 10’, ‘more than 10’ and ‘don’t know’); sunburn events in last 6 weeks (‘once’, ‘2-10 times’, ‘don’t know’); and, bad sunburns (‘one’, ‘2 to 10’ and ‘don’t know’). Data from participants who did not complete follow-up data was included if surveys were completed.

Multimedia Appendix Tables, Figure and Results

Table S1. Skin sensitivity, tanning responses, number of moles and freckles of participants upon recruitment at baseline.

	Community phase 1 pilot (n)		*Community phase 2 pilot* (n)		*School pilot* (n)		**Combined**	
**Intervention group**	Placebo	Test	Placebo	Test	Placebo	Test[b]	Placebo	Test
**Participants completing baseline** (n)[a]	8	8	12	12	8	8	28	28
**Skin reaction to 30 minutes of sun exposure**[c]								
Not get sunburnt at all	0	1	3	1	5	8	8 (29)	10 (36)
Get mildly sunburnt	6	5	7	8	3	0	16 (57)	13 (46)
Have painful sunburn	1	1	1	3	0	0	2 (7)	4 (14)
Get severe sunburn with blistering	1	1	0	0	0	0	1 (4)	1 (4)
Uncertain of response/don't know	0	0	1	0	0	0	1 (4)	0 (0)
**After this initial reaction, would you get a tan?**								
Yes	5	5	4	8	6	7	15 (54)	20 (71)
No	3	3	5	3	2	1	10 (36)	7 (25)
Uncertain of response/don't know	0	0	3	1	0	0	3 (11)	1 (4)
**Skin appearance at the end of summer**[d]								
No suntan at all	2	4	0	3	0	1	2 (7)	8 (29)
Lightly tanned	5	3	4	1	4	1	13 (46)	5 (18)
Moderately tanned	1	1	8	5	3	3	12 (43)	9 (32)
Very tanned	0	0	0	3	1	2	1 (4)	5 (18)
**Number of moles on skin**								
No moles	1	1	3	3	2	1	6 (21)	5 (18)
A few moles (less than 10 over entire body)	4	6	5	9	6	5	15 (54	20 (71)
Some moles (less than 50 over entire body)	2	0	4	0	0	1	6 (21)	1 (4)
Many moles	1	1	0	0	0	1	1 (4)	2 (7)
**Number of freckles on face at end of summer**								
No freckles	1	1	5	2	3	5	9 (32)	8 (29)
A few freckles	4	6	5	7	4	3	13 (46)	16 (57)
Some freckles	2	0	2	1	1	0	5 (18)	1 (4)
Many freckles	1	1	0	2	0	0	1 (4)	3 (11)

Data are shown as number (n) of each participant who selected each response, except for combined data which also shows percentage (%) with each intervention group (i.e., n (%))

[a] Participants recruited into each pilot who completed all baseline questionnaires and were then given access to either the placebo (*SunDial*) or test (*Sun Safe*) apps for 6 weeks

[b] One test participant did not complete baseline surveys as they were not able to attend the in-school session

[c] Skin reaction to 30 min of sun in middle of day without wearing sunscreen for the first time in summer

[d] Skin appearance at the end of summer after spending short periods of time in the sun, without sunscreen, every day over summer

For data combined across the 3 pilot studies, statistical comparisons were made between placebo and test interventions using the Fisher's Exact (with groups collapsed as described in methods above):

- skin reaction to 30 min of sun exposure, relative risk (RR)=0.8 (95% confidence interval (CI), 0.4, 1.7), *P* =.77
- would you get a tan? RR=0.8 (95%CI, 0.5, 1.1), *P*=.27
- skin appearance at end of summer, RR=1.1 (95%CI, 0.7, 1.9), *P*=.79
- number of moles on skin, RR=1.2 (95%CI, 0.4, 3.4), *P* >.99
- number of freckles on skin, RR=1.1 (95%CI, 0.5, 2.5), *P* >.99

Table S2. Data related to downloading of the apps and frequency of their use.

	Community phase 1 pilot		*Community phase 2 pilot*		*School pilot*		**Combined**	
**Intervention group**[a]	Placebo	Test	Placebo	Test[b]	Placebo	Test	Placebo	Test
**Participants** (n)	8	8	12	12	8	9	28	29
**Confirmed app was download** (n)	4	8	12	8	N/A		16	16
**Days taken to download app**[c] (mean (SD))	2.3 (1.5)	5.0 (7.3)	4.6 (2.6)	3.6 (1.8)	N/A		4.0 (2.6)	4.3 (5.2)
**Days of access to app**[d] (mean (SD))	44 (2)	47 (6)	42 (7)	43 (3)	N/A		43 (6)	45 (5)
**Number completing study** (n)	7	7	11	9	8	9	26	25
**Device used to access app**								
Smartphone only	3	6	6	4	6	4	15 (58)	14 (56)
Tablet only	2	1	5	3	2	4	9 (35)	8 (32)
Smartphone and tablet	1	0	0	1	0	0	1 (4)	1 (4)
Prefer not to say/no response	1	0	0	1	0	1	1 (4)	2 (8)
**Frequency of app use**								
Everyday	0	1	0	2	0	0	0 (0)	3 (12)
Most days (2-3 times a week)	1	2	0	1	1	0	2 (8)	3 (12)
Once a week	3	0	4	2	0	0	7 (27)	2 (8)
Once a fortnight	1	1	3	1	3	5	7 (27)	7 (28)
Once or twice (in total)	1	3	4	2	2	3	7 (27)	8 (32)
Not at all	1	0	0	1	2	1	3 (12)	2 (8)
**Use on days when accessed**[e]								
Once	2	2	9	6	7	5	18 (69)	13 (52)
2-5 times	2	5	1	2	1	3	4 (15)	10 (40)
5-10 times	1	0	1	0	0	0	2 (8)	0 (0)
Prefer not to say/no response	2	0	0	1	0	2	2 (8)	2 (8)

Data are shown as number (n) of each participant who selected each response, except when combined which also shows number and percentage (i.e., n (%)) within each intervention group, and for 'Days taken to download app' and 'Days of access to app', which are shown as mean (SD). N/A = not applicable as data were not collected during recruitment and throughout School pilot at an individual level as most participants downloaded these app during an in-class session, and because of governance constraints that prevented researchers from contacting participants out of in-school sessions.

[a] Participants recruited into each pilot were given access to either the placebo (*SunDial*) or test (*Sun Safe*) apps for 6 weeks

[b] One participant stated they had issues downloading the app

[c] Days taken to download app from date initial email requesting app download was sent to the date the participant acknowledged they had downloaded the app (inclusive) via email/SMS

[d] Days participants had access to either app from the date they confirmed they had downloaded the app to the date participants completed all post-app questionnaires (inclusive)

[e] Frequency of use of app on days when accessed

For data combined across the 3 pilot studies, statistical comparisons were made between placebo and test interventions using the Mann-Whitney tests for days taken to download (P=.64) and days of access to app (P=.62)

Table S3. Correctly answered multiple-choice knowledge quiz questions.

Q#		*Before intervention*		*During intervention*	
	**Intervention group**	Placebo	Test	Placebo	Test
	**Number of participants** (n)	28	30	25	25
**Q#**	**Questions**				
Q1	Sunburn increases the risk of which cancer?	28 (100)	30 (100)	25 (100)	24 (96)
		n/a		[RR=1.0 (95%CI, 0.9, 1.1), *P*=.31]	
Q2	Which wavelengths of sunlight are most risky for sunburn, and for making beneficial vitamin D in your skin?	12 (43)	11 (37)	11 (44)	10 (40)
		[RR=1.2 (95%CI, 0.6, 2.2), *P*=.63]		[RR=1.1 (95%CI, 0.6, 2.1), *P*=.77]	
Q3	True or False? Solstice events occur in autumn and spring.	14 (50)	17 (57)	13 (52)	15 (60)
		[RR = 0.9 (95%CI, 0.5, 1.4), *P*=.61]		[RR=0.9 (95%CI, 0.5, 1.4), *P*=.57]	
Q4	At which UV index values are sun protection recommended when you are outside?	14 (50)	15 (50)	13 (52)	20 (80)
		[RR=1.0 (95%CI, 0.6, 1.7), *P*>.99]		**[RR=0.65 (95%CI, 0.41, 0.97), *P*=.04]**	
Q5	Which cancer is the most common cancer in young adults in Australia?	18 (64)	20 (67)	18 (72)	17 (68)
		[RR=0.96 (95%CI, 0.6, 1.4), *P*=.85]		[RR=1.1 (95%CI, 0.7, 1.6), *P*=.76]	
Q6	The equinox is the time(s) or date(s) at which the sun crosses the celestial equator. How many times a year does this occur?	19 (68)	16 (53)	13 (52)	19 (76)
		[RR=1.3 (95%CI, 0.8, 2.0), *P*=.26]		[RR=0.7 (95%CI, 0.4, 1.0), *P*=.08]	
Q7	What is the best definition of the UV index?	20 (71)	21 (70)	21 (84)	19 (76)
		[RR=1.0 (95%CI, 0.7, 1.4), *P*>.99]		[RR=1.1 (95%CI, 0.8, 1.5), *P*=.73]	
Q8	Which of these factors does NOT affect the UV index?	22 (79)	26 (87)	14 (56)	20 (80)
		[RR=0.9 (95%CI, 0.7, 1.2), *P*=.31]		[RR=0.7 (95%CI, 0.4, 1.0), *P*=.07]	
Q9	True or False: There are other benefits of sun exposure than vitamin D.	23 (82)	25 (83)	22 (88)	22 (88)
		[RR=0.99 (95%CI, 0.8, 1.3), *P*=.90]		[RR=1.0 (95%CI, 0.8, 1.3), *P*>.99]	
Q10	Too much sun exposure may cause:	24 (86)	27 (90)	25 (100)	22 (88)
		[RR=0.95 (95%CI, 0.7, 1.2), *P*=.62]		[RR=1.1 (95%CI, 0.98, 1.2), *P*=.07]	
Q11	True or False: People with fair skin are at increased risk of skin cancer compared to those with dark skin.	25 (89)	24 (80)	23 (92)	24 (96)
		[RR=1.1 (95%CI, 0.9, 1.4), *P*=.33]		[RR=0.96 (95%CI, 0.8, 1.2), *P*=.55]	
Q12	When you need sun protection outside, you should:	27 (96)	30 (100)	25 (100)	25 (100)
		[RR=1.0 (95%CI, 0.8, 1.1), *P*=.30]		n/a	
Q13	Does shade provide complete protection from UV radiation?	27 (96)	29 (97)	24 (96)	25 (100)
		[RR=1.0 (95%CI, 0.8, 1.2), *P*=.96]		[RR=0.96 (95%CI, 0.8, 1.1), *P*=.31]	
Q14	Where does Western Australia rank in terms of rates of skin cancer among Australian states and territories?	10 (36)	14 (47)	11 (44)	17 (68)
		[RR = 0.8 (95%CI, 0.4, 1.4), *P*=.40]		[RR=0.6 (95%CI, 0.4, 1.1), *P*=.09]	
Q15	True or False: Oily fish are a good dietary source of vitamin D.	15 (54)	20 (67)	17 (68)	17 (68)
		[RR=0.8 (95%CI, 0.5, 1.2), *P*=.31]		[RR=1.0 (95%CI, 0.7, 1.5), *P*>.99]	
Q16	When you need sun protection outside, broad-spectrum 30+ SPF sunscreen should be applied at least:	18 (64)	18 (60)	16 (64)	18 (72)
		[RR=1.1 (95%CI, 0.7, 1.6), *P*=.74]		[RR=0.9 (95%CI, 0.6, 1.3), *P*=.54]	
Q17	When does the vernal equinox occur?	6 (21)	8 (27)	9 (36)	11 (44)
		[RR=0.8 (95%CI, 0.3, 2.0), *P*=.64]		[RR=0.8 (95%CI, 0.4, 1.6), *P*=.56]	
Q18	True or false: You can make vitamin D while driving in the car if your skin is exposed to sunlight coming through the window glass.	4 (14)	7 (23)	7 (28)	8 (32)
		[RR=0.6 (95%CI, 0.2, 1.8), *P*=.38]		[RR=0.9 (95%CI, 0.4, 2.0), *P*=.76]	
Q19	Select the missing words. *It is necessary to expose ___________ to sunlight to produce vitamin D.*	26 (93)	30 (100)	25 (100)	25 (100)
		[RR=0.9 (95%CI, 0.8, 1.1), *P*=.14]		n/a	

Q20	True or False: Tanning increases your risk of skin cancer.	22 (79)	26 (87)	22 (88)	20 (80)
		[RR=0.9 (95%CI, 0.7, 1.2), *P*=.41]		[RR=1.1 (95%CI, 0.8, 1.5), *P*=.44]	

Data are shown combined for the number (n) of each participant and percentage (%) within each intervention who answered each question correctly, acquired from participants enrolled in one of three pilot studies, who completed the quiz before and after 6 weeks of access to either the placebo (*SunDial*) or test (*Sun Safe*) apps (i.e., n (%)). A Chi-squared test was used to test for statistical differences in questions answered correctly between placebo and test intervention groups, with results shown as: [relative risk (RR)=1.0 (95% confidence interval (CI)), *P*-value] (n/a = test not applicable).

Sun health knowledge did not differ by gender

There was no difference between males and females in the percentage of correct answers achieved before (male = 65.0 ± 10.8%; female = 68.9 ± 9.8%; 3.9% (95%CI, -2.0, 9.8), *P*=.19, 2-way ANOVA) or after (male = 72.5 ± 10.9%; female =72.4 ± 10.7%; -0.1% (95%CI, -6.8, 6.5), *P*=.97) the intervention. There was also no difference between intervention groups in the time taken to complete the quiz either before (placebo = 5.5 ± 4.4 min; test = 4.4 ± 1.7 min; 0.8 min (95%CI, -1.0, 2.5), *P*=.38, 2-way ANOVA) or after (placebo = 5.0 ± 5.6 min; test = 4.4 ± 2.3 min; 0.4 min (95%CI, -1.9, 2.8), *P*=.71) the intervention. Doing the quiz previously did not significantly reduce the time taken to complete it (before = 4.8 ± 3.3 min; after = 4.7 ± 4.3 min; 0.1 min (95%CI, -1.4, 1.5), *P*=.92, 2-way ANOVA).

Table S4. Time spent outdoors (minutes) per day in the 6 weeks before or during the intervention.

	Community phase 1 pilot				*Community phase 2 pilot*				*School pilot*			
	**Before**		**During**		**Before**		**During**		**Before**		**During**	
**Intervention group**	Placebo[a]	Test	Placebo	Test[b]	Placebo[c]	Test	Placebo	Test	Placebo	Test	Placebo	Test
**Number of participants**, n	8	8	7	6	12	12	11	9	8	8	8	9
**Overall**	260 (76)	239 (100)	148 (64)	146 (71)	219 (117)	164 (65)	174 (115)	214 (102)	143 (75)	114 (38)	132 (49)	147 (91)
	[*P*=.45]		[*P*=.96]		[*P*=.88]		[*P*=.22]		[*P*=.34]		[*P*=.81]	
**Weekdays during school week**	238 (72)	244 (114)	148 (68)	185 (60)	224 (192)	171 (61)	185 (127)	165 (44)	128 (60)	114 (41)	111 (38)	133 (58)
	[*P*=.91]		[*P*=.11]		[*P*=.38]		[*P*=.70]		[*P*=.61]		[*P*=.36]	
**Weekend days**	292 (125)	227 (110)	152 (79)	160 (103)	209 (207)	145 (92)	187 (148)	224 (156)	197 (121)[d]	118 (61)	99 (75)	153 (146)
	[*P*=.28]		[*P*=.88]		[*P*=.35]		[*P*=.51]		[*P*=.13]		[*P*=.52]	
**School holidays (days at home)**	N/A	N/A	145 (56)	139 (88)	N/A	N/A	132 (105)	193 (140)	N/A	N/A	122 (88)	178 (128)
			[*P*=.88]				[*P*=.28]				[*P*=.38]	

Data are shown as mean (SD) in minutes, with n = number of participants and p-values shown in [ ] the results of Student's t/Mann-Whitney tests comparing placebo and test groups within each pilot study (before or after intervention)

[a] One placebo participant was on school holidays and a different placebo participant was at another location (Brisbane, QLD) during the 6 weeks before the intervention commenced

[b] One participant did not provide sufficient information for these data to be calculated

[c] Two participants were home schooled for some or all this time

[d] One participant did not provide a response to weekends during school week (n=7 for this time)

N/A = not applicable as no school holidays occurred in this phase of pilot studies

For data combined from the 3 pilot studies, 2-way ANOVA (with Tukey's post-hoc) was used to test for statistical differences in time spent outdoors between placebo and test intervention groups:

- overall (before), 20 min difference in predicted means (95% confidence interval (CI), -18, 96), *P*=.17
- overall (during), -18 min (95% CI, -68, 33), *P*=.33
- weekdays (before), 20 min (95% CI, -40, 80), *P*=.51
- weekdays (during), -13 min (95% CI, -51, 31), *P*=.56
- weekend days (before), 69 min (95% CI, -5, 143), *P*=.07
- weekdays (during), -33 min (95% CI, -107, 41), *P*=.37
- school holidays (during), -37 min (95% CI, -98, 24), *P*=.23

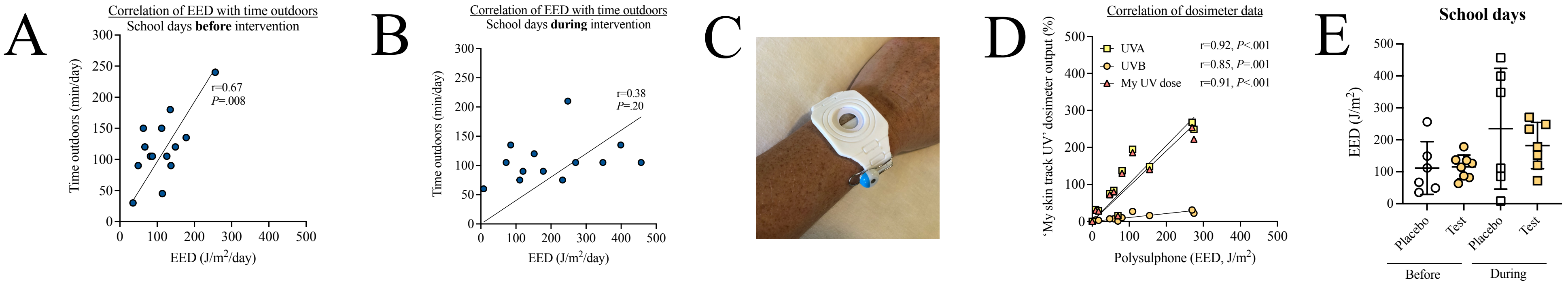

Figure S1. *Dosimeter-based measurement of UV exposure in participants of the School pilot.* The erythemally effective doses (EED, J/m²) on school days received by participants of the School pilot who returned polysulphone sun badges worn in the week before the intervention commenced and in the last week of the intervention are shown with linear correlations (Pearson's test) between EED and time outdoors before (A, n=14) and during (B, n=13) the intervention. In (C), a photograph of the wrist of the researcher who wore the polysulphone (white wristband, with sun badge insert) and 'My Skin Track UV' (blue clip-on) dosimeters. In (D), the linear correlations (Pearson's test) between EED (from polysulphone dosimeter) with UVA, UVB and 'My UV dose' outputs (%) by the 'My Skin Track UV' dosimeter (n=12 days). N.B. On two days, data collection using the 'My Skin Track UV' failed due to synchronicity problems between the dosimeter and its app. In (E), the EED on school days received by participants of the School pilot in the week before the intervention commenced ('Before'; n=6 for placebo, n=8 for test, *P*=.91, Student's t) and the last week of the intervention ('During'; n=6 for placebo, n=7 for test, *P*=.54, Student's t) with data shown as mean ± SD.

Validation of time outdoors with dosimetry data. A researcher volunteered to wear polysulphone dosimeters at the same times as participants (for 14 days) as well as an alternative dosimeter, 'My Skin Track UV' (La Roche-Posey, Multimedia Appendix Figure 1C). EED levels measured using the polysulphone dosimeters significantly correlated with both UVA (Pearson's r=0.92, *P*<.001) and UVB (Pearson's r=0.85, *P*=.001) outputs reported by the 'My Skin Track UV' dosimeter (Multimedia Appendix Figure 1D).

Compliance in wearing UV dosimeters (sun badges). Personalised UV exposure was measured by dosimeters (sun badges) in the School pilot. 'Dosimeter diaries' were developed as a means of measuring compliance in the wearing of UV dosimeters and were a useful tool in validating negative datapoints. Indeed, for nearly all days that the dosimeters were not worn, erythemally effective doses (EED) (measured by polysulphone dosimeters, J/m^2) [27] were below the limit of detection (<10 J/m^2) (Table S5). Most participants wore the sun badges on weekdays (compliance >70% across the study), with reduced compliance on weekend days particularly in the last week of the intervention (≤33%) with limited difference between interventions (Table S5). Reasons given by participants for not wearing badges included: "I forgot'"; "I was in water all day"; "I was inside all day"; or because of an event (e.g., "a festival").

Table S5. Sun badge compliance outcomes.

	In the week before intervention		In the last week of the intervention	
**Intervention group**	Placebo	Test	Placebo	Test
**Number of participants**, n	8	9	8	9
**Sun badge packs returned**, n (%)	6 (75)	8 (89)	6 (75)	7 (78)
**Sun badge diary completed**, n (%)	4 (50)	7 (78)	6 (75)	7 (78)
**Number of weekdays participants stated they wore badges**[a]	19 days (95% of possible days)	35 days (100% of possible days)	22 days (73% of possible days)	28 days (80% of possible days)
**Weekend days participants said they wore badges**[a]	8 days (100% of possible days)	10 days (71% of possible days)	4 days (33% of possible days)	3 days (21% of possible days)
**Times when badges not worn and measured EED was below LOD**, n (%)[b]	0 (0)	4 (100)	26 (96)[c]	17 (94)[d]

[a] Of those who completed the sun badge diary
[b] LOD = limit of detection was <10 J/m^2, EED = erythemally effective doses
[c] One badge was missing
[d] Value was still 'low' (15 J/m^2) for the 1 dosimeter reading above the LOD

Table S6. Sunscreen use.

	Before intervention		*During intervention*	
**Intervention group**	Placebo	Test	Placebo	Test
**Number of participants** (n)	28	28	26	25
**Sunscreen application on school days**				
Never/infrequently	19 (68)	19 (68)	14 (54)	14 (56)
Sometimes/Always (everyday)	9 (32)	9 (32)	12 (46)	11 (44)
**Time of application on school days**[a]				
Morning or afternoon	8 (89)	8 (89)	8 (67)	9 (82)
Morning and afternoon	1 (11)	1 (11)	4 (33)	2 (18)
**Sunscreen application on non-school days**				
Never/infrequently	18 (64)	17 (61)	11 (42)	15 (60)
Sometimes/Always (everyday)	10 (36)	11 (39)	15 (58)	10 (40)
**Time of application on non-school days**[a]				
Morning or afternoon	4 (40)	8 (73)	8 (53)	7 (70)
Morning and afternoon	6 (60)	3 (27)	7 (47)	3 (30)
**Attendance at special outdoor events**[b]				
Yes	19 (68)	17 (61)	17 (65)	16 (64)
No	9 (32)	11 (39)	9 (35)	9 (36)
**Did you apply sunscreen at this special event?**				
Yes	10 (53)	11 (65)	13 (76)	13 (81)
No	9 (47)	5 (29)	4 (24)	3 (19)
No response	0 (0)	1 (6)	0 (0)	0 (0)
**Time of application for special outdoor event**[a]				
Morning or afternoon	4 (40)	9 (82)	6 (46)	9 (69)
Morning and afternoon	6 (60)	2 (18)	7 (54)	4 (31)

Combined data are shown as number (n) of each participant and percentage (%) within each intervention who selected each response, acquired from participants enrolled in one of three pilot studies, who completed the survey before and after 6 weeks of access to either the placebo (*SunDial*) or test (*Sun Safe*) apps (i.e., n (%))

[a] For those who sometimes or always applied sunscreen or said 'yes' to applying sunscreen

[b] Question: In the last 6 weeks, did you attend a special event outdoors that was not for school (e.g., went to the beach)?

A Fisher's Exact test was used to test for statistical difference in sunscreen application between placebo and test intervention groups:

- on school days (before), relative risk (RR)=1.0 (95% confidence interval (CI), 0.7, 1.5), P>.99
- on school days (during), RR=1.0 (95% CI, 0.6, 1.6), P>.99
- on non-school days (before), RR=1.1 (95% CI, 0.7, 1.6), P>.99
- on non-school days (during), RR=0.7 (95% CI, 0.4, 1.2), P=.27
- at a special event (before), RR=0.6 (95% CI, 0.2, 1.1), P=.20 (excluding 'No response')
- at a special event (during), RR=0.9 (95% CI, 0.6, 1.4), P>.99

Table S7. Sun protective behaviours on school days while outdoors between 10 AM and 3 PM.

	Before intervention		***During intervention***	
**Intervention group**	Placebo[a]	Test[a]	Placebo[b]	Test[c]
**Number of participants** (n)	27	27	26	24
**Wearing a hat**				
<50% of the time	18 (67)	21 (78)	20 (77)	16 (67)
≥50% of the time	9 (33)	6 (22)	6 (23)	8 (33)
**Wearing clothing with long sleeves**				
<50% of the time	16 (59)	11 (41)	19 (73)	20 (83)
≥50% of the time	11 (41)	16 (59)	7 (27)	4 (17)
**Wearing full-length trousers or long skirt**				
<50% of the time	12 (44)	21 (78)	19 (73)	22 (92)
≥50% of the time	15 (56)	6 (22)	7 (27)	2 (8)
**Spend time in the shade**				
<50% of the time	10 (37)	12 (44)	8 (32)	6 (25)
≥50% of the time	17 (63)	15 (56)	17 (68)	18 (75)

Combined data are shown as number (n) of each participant and percentage (%) within each intervention who selected each response, acquired from participants enrolled in one of three pilot studies, who completed the survey before and after 6 weeks of access to either the placebo (*SunDial*) or test (*Sun Safe*) apps (i.e., n (%)).

[a] One participant from each arm did not answer these questions before the intervention as they did not spend any time outdoors

[b] One participant answered 'X' for spending time in the shade during the intervention, and this result was excluded

[c] One participant did not answer these questions for during the intervention as no time was spent outdoors

A Fisher's Exact test was used to test for statistical differences between placebo and test intervention groups in:

- wearing a hat (before), relative risk (RR)=0.9 (95% confidence interval (CI), 0.6, 1.2), *P*=.54
- wearing a hat (during), RR=1.2 (95% CI, 0.8, 1.7), *P*=0.53
- wearing clothing with long sleeves (before), RR=1.5 (95% CI, 0.8, 2.6), *P*=0.28
- wearing clothing with long sleeves (during), RR=0.88 (95% CI, 0.6, 1.2), *P*=0.50
- wearing full-length trousers or long skirt (before), **RR=0.6 (95% CI, 0.3, 0.9), *P*=.02**
- wearing full-length trousers or long skirt (during), RR=0.8 (95% CI, 0.6, 1.0), *P*=.14
- spend time in the shade (before), RR=0.8 (95% CI, 0.4, 1.6), *P*=.78
- spend time in the shade (during), RR=1.3 (95% CI, 0.5, 3.1), *P*=.75

Table S8. Mean ratings across each area of assessment in uMARS survey by participants of pilot studies.

	Community phase 1 pilot			*Community phase 2 pilot*			*School pilot*			**Combined**		
**Intervention group**	Placebo		Test	Placebo		Test	Placebo		Test[b]	Placebo		Test
**Number of participants** (n)	5-7		9-11	9-11		9	5-8		7-8	19-26		23-24
**Areas of assessment**[a]												
Engagement	3.2 (0.9)	[*P*=.85]	3.2 (0.9)	2.1 (0.8)	[*P*=.13]	2.6 (0.5)	1.9 (0.6)	**[*P*=.001]**	2.9 (0.4)	2.3 (0.9)	**[-0.5 (-1.0, -0.1) *P*=.01]**	2.9 (0.6)
Functionality	3.7 (0.9)[c]	[*P*=.98]	3.8 (0.9)	2.8 (0.6)	**[P<.001]**	4.0 (0.6)	2.9 (0.9)[e]	**[*P*=.02]**	3.9 (0.2)	3.1 (0.9)	**[-0.7 (-1.2, -0.3) *P*<.001]**	3.9 (0.6)
Aesthetics	4.1 (0.7)	[*P*=.74]	4.2 (0.8)	3.4 (0.7)	[*P*=.68]	3.6 (0.9)	3.3 (1.1)	[*P*=.23]	3.8 (0.6)	3.6 (0.9)	[0.3 (-0.7, 0.2) *P*=.23]	3.9 (0.8)
Information	3.6 (1.0)	**[*P*=.006]**	4.5 (0.3)[d]	2.8 (0.8)[c]	**[*P*=.01]**	3.8 (0.8)	3.5 (0.5)[e]	**[*P*=.02]**	4.3 (0.5)[d]	3.2 (0.8)	**[-0.9 (-1.4, -0.5) *P*<.001]**	4.2 (0.6)
Overall quality[f]	3.5 (0.9)	[*P*=.24]	3.8 (0.8)	2.7 (0.5)	**[*P*=.009]**	3.5 (0.6)	3.1 (0.4)	**[*P*=.005]**	3.8 (0.3)	3.0 (0.9)	**[-0.7 (-1.2, -0.2) *P*=.01]**	3.7 (0.8)
Subjective quality	3.2 (1.4)	[*P*=.30]	3.9 (1.2)	2.4 (1.0)	[*P*=.09]	3.2 (0.6)	2.0 (0.9)	**[*P*=.02]**	3.0 (0.6)	2.5 (1.2)	**[-0.9 (-1.5, -0.3) *P*=.006]**	3.3 (0.9)
Perceived impact	3.2 (1.4)	[*P*=.78]	3.5 (1.1)	2.4 (1.1)	**[*P*=.02]**	3.6 (0.8)	2.0 (1.1)	**[*P*=.02]**	3.2 (0.6)	2.5 (1.2)	**[-0.8 (-1.4, -0.2) *P*=.006]**	3.4 (0.8)

Data are shown as mean (SD) for participants enrolled in one of three pilot studies, who completed the survey after 6 weeks of access to either the placebo (*SunDial*) or test (*Sun Safe*) apps. Student's t tests (with Welsh correction if required) or Mann-Whitney tests were performed to compare between placebo and test groups within each pilot study, depending on whether data were normally distributed with p-values shown in [ ]. When data were combined for all pilot studies, all were normally distributed (by Shapiro-Wilk normality tests), with 2-way ANOVA used to compare data between placebo and test groups, with differences in predicted mean scores (95% confidence interval, CI), and *P*-values shown in [ ].

[a] Mean score for each area of assessment were determined for each participant across items within each area that included assessments that used a 5-point Likert scale for participants who completed all items for each area.

[b] One participant in this group did not complete any of the questions related on any of the 25 questions posed related to the uMARS areas of engagement. This participant had trouble downloading the *Sun Safe* app, which was likely due to the use of an older iOS device which did not support the app.

[c] Questions were not answered by 2 participants

[d] Questions were not answered by 1 participant

[e] Questions were not answered by 3 participants

[f] 'Overall quality' was calculated from the mean score of the Engagement, Functionality, Aesthetics and Information areas of assessment, and was only calculated for participants who completed all questions in the uMARS survey.

Table S9. Assessment of subjective quality of apps by participants of pilot studies.

	Community phase 1 pilot		*Community phase 2 pilot*		*School pilot*		**Combined**	
**Intervention group**	Placebo	Test	Placebo	Test	Placebo	Test	Placebo	Test
**Number of participants** (n)	7	7	11	9	8	8	26	24
**Items**[a]								
Would you recommend this app to people who might benefit from it? (mean (SD))	3.3 (1.5)	4.0 (1.5)	2.3 (0.9)	2.9 (0.8)	1.5 (0.8)	3.1 (0.6)	2.3 (1.2)	3.3 (1.1)
	[*P*=.37]		[*P*=.13]		**[*P*=.003]**		**[*P*=.003]**	
How many times do you think you would use this app in the next 12 months? (mean (SD))[b]	3.0 (1.5)	4.0 (1.4)	2.4 (1.4)	3.7 (0.7)	2.1 (1.1)	2.8 (0.9)	2.5 (1.3)	3.5 (1.1)
	[*P*=.19]		**[*P*=.02]**		[*P*=.24]		**[*P*=.008]**	
Would you pay for this app? (n (%))								
Yes	2 (29)	2 (29)	0 (0)	0 (0)	0 (0)	1 (12)	2 (8)	3 (12)
No	5 (71)	5 (71)	11 (100)	9 (100)	8 (100)	7 (78)	24 (92)	21 (88)
							[RR=0.6 (95%CI, 0.1, 2.9), *P*=.66]	

Data are shown for participants enrolled in one of three pilot studies, who completed the survey after 6 weeks of access to either the placebo (*SunDial*) or test (*Sun Safe*) apps. A student's t test (with Welsh correction if required) or Mann-Whitney test or Fisher's Exact test (for the 'Would you pay for this app' item only, combined data) was performed to compare between placebo and test groups within each pilot study (depending on whether data were normally distributed). When data were combined for all pilot studies, none were normally distributed (as determined by Shapiro-Wilk normality tests), and so a Mann-Whitney test was used to compare data if continuous. *P*-values are shown in [ ], with RR = relative risk, and CI = confidence interval shown for 'Would you pay for this app' data.

[a] Across 5 questions, participants rated the 'subjective quality' of the app they used on a 5-point Likert scale (except for the Yes/No response for the 'Would you pay for this app?' item)

[b] The 5-point Likert scale for this question was: none, 1-2 times, 3-10 times, 10-50 times, >50 times (1-5, respectively)
